# Oncogenic RABL6A promotes NF1-associated MPNST progression in vivo

**DOI:** 10.1093/noajnl/vdac047

**Published:** 2022-04-09

**Authors:** Jordan L Kohlmeyer, Courtney A Kaemmer, Joshua J Lingo, Ellen Voigt, Mariah R Leidinger, Gavin R McGivney, Amanda Scherer, Stacia L Koppenhafer, David J Gordon, Patrick Breheny, David K Meyerholz, Munir R Tanas, Rebecca D Dodd, Dawn E Quelle

**Affiliations:** 1 Molecular Medicine Graduate Program, The University of Iowa, Iowa City, Iowa, USA; 2 Cancer Biology Graduate Program, The University of Iowa, Iowa City, Iowa, USA; 3 Medical Scientist Training Program, The University of Iowa, Iowa City, Iowa, USA; 4 The Department of Neuroscience and Pharmacology, The University of Iowa, Iowa City, Iowa, USA; 5 The Department of Pathology, The University of Iowa, Iowa City, Iowa, USA; 6 The Department of Internal Medicine, The University of Iowa, Iowa City, Iowa, USA; 7 The Department of Pediatrics, The University of Iowa, Iowa City, Iowa, USA; 8 Department of Biostatistics, The University of Iowa, Iowa City, Iowa, USA; 9 The Holden Comprehensive Cancer Center, The University of Iowa, Iowa City, Iowa, USA

**Keywords:** MPNST, Myc, NF1, RABL6A, YAP

## Abstract

**Background:**

Malignant peripheral nerve sheath tumors (MPNSTs) are aggressive sarcomas with complex molecular and genetic alterations. Powerful tumor suppressors *CDKN2A* and *TP53* are commonly disrupted along with *NF1*, a gene that encodes a negative regulator of Ras. Many additional factors have been implicated in MPNST pathogenesis. A greater understanding of critical drivers of MPNSTs is needed to guide more informed targeted therapies for patients. RABL6A is a newly identified driver of MPNST cell survival and proliferation whose *in vivo* role in the disease is unknown.

**Methods:**

Using CRISPR-Cas9 targeting of *Nf1 + Cdkn2a* or *Nf1 + Tp53* in the mouse sciatic nerve to form *de novo* MPNSTs, we investigated the biological significance of RABL6A in MPNST development. Terminal tumors were evaluated by western blot, qRT-PCR, and immunohistochemistry.

**Results:**

Mice lacking *Rabl6* displayed slower tumor progression and extended survival relative to wildtype animals in both genetic contexts. YAP oncogenic activity was selectively downregulated in *Rabl6*-null, *Nf1 + Cdkn2a* lesions whereas loss of RABL6A caused upregulation of the CDK inhibitor, p27, in all tumors. Paradoxically, both models displayed elevated Myc protein and Ki67 staining in terminal tumors lacking RABL6A. In *Nf1 + p53* tumors, cellular atypia and polyploidy were evident and increased by RABL6A loss.

**Conclusions:**

These findings demonstrate that RABL6A is required for optimal progression of NF1 mutant MPNSTs *in vivo* in both *Cdkn2a* and *p53* inactivated settings. However, sustained RABL6A loss may provide selective pressure for unwanted alterations, including increased Myc, cellular atypia, and polyploidy, that ultimately promote a hyper-proliferative tumor phenotype akin to drug-resistant lesions.

**Key Points**

- The RABL6A oncoprotein promotes NF1-associated MPNST progression *in vivo*.
- Myc upregulation may mediate refractory MPNST growth.
- Advanced malignancy of *Nf1 + p53* (vs *Nf1 + Cdkn2a*) altered MPNSTs in C57BL/6 mice.

**Importance of the Study**

MPNSTs are deadly tumors that lack effective therapies. Many factors implicated in MPNST genesis have yet to be fully tested for biological significance in disease formation. We establish a critical physiological role for a new oncoprotein, RABL6A, in promoting NF1-associated MPNST progression. We identify novel RABL6A-regulated pathways that likely contribute to tumor growth, specifically YAP and Myc signaling, and found that sustained RABL6A loss eventually yields more proliferative tumors. We liken RABL6A deficient tumors to those being treated with therapies targeting RABL6A effectors, such as CDKs, providing a powerful platform to uncover mediators of drug resistance. Our data suggest oncogenic Myc may mediate refractory MPNST growth. Notably, *Nf1 + p53* inactive MPNSTs displayed features of advanced malignancy compared to *Nf1 + Cdkn2a* deficient tumors in C57BL/6 mice, linking tumor aggressiveness to *p53* versus *Cdkn2a* loss. This study highlights a novel *in vivo* system to directly compare different mutational combinations in MPNST pathogenesis.

Malignant peripheral nerve sheath tumors (MPNSTs) are deadly soft tissue sarcomas that arise from the Schwann cells surrounding peripheral nerves.^1^ MPNSTs occur sporadically or in association with the tumor-prone, neurological disorder Neurofibromatosis Type I (NF1). Disruption of the *NF1* gene, which encodes neurofibromin, a negative regulator of the Ras oncoprotein, is a key underlying feature of MPNST pathogenesis.^2^ In NF1 disease, loss of neurofibromin induces benign neurofibromas (NFs) that can undergo stepwise malignant transformation following loss of the *INK4A/ARF* (also called *CDKN2A*) and/or *TP53* tumor suppressors.^1,3^ A number of other factors have been implicated in MPNST development; however, many of them still require biological testing.^4^ Thus, while our understanding of MPNST development is continually expanding, much more remains to be learned about the critical alterations driving disease progression. Elucidating these pathways will guide the rational development of targeted therapies to combat MPNSTs, which currently lack effective treatments.

To uncover meaningful alterations driving MPNSTs, genetically engineered mouse models (GEMMs) have been generated to delineate genes and pathways contributing to MPNST biology. Some of the earliest GEMMs to successfully develop MPNSTs involved combined loss of *Nf1* with *Trp53*,^5,6^*Pten,*^7^ or *Cdkn2a*.^8,9^ Others employed *Sleeping Beauty* transposon-based mutagenesis in p53 mutant mice with overexpressed EGFR to identify cooperating mutations in MPNST pathogenesis.^10^ Alterations in Wnt/β-catenin, PI3K-AKT-mTOR, and growth factor receptor signaling pathways were found to promote tumorigenesis in concert with p53 loss and EGFR activation. Most recently, delivery of adenoviruses containing Cas9 and single-guide RNAs (sgRNAs) targeting *Nf1* with *p53* into the sciatic nerve of wildtype mice was found to efficiently induce primary MPNSTs.^11^ The model produced lesions that are remarkably similar, both genetically and histologically, to human MPNSTs. This provided a unique, rapid, and cost-effective tool for investigating MPNST biology. Not only can additional gene targets be concurrently altered by including more sgRNAs in the adenoviral Cas9 construct, but CRISPR editing of *Nf1 + p53* can be conducted in the peripheral nerves of mice with distinct genotypes.

We recently identified RABL6A, an oncogenic GTPase, as a new regulator of MPNST pathogenesis.^12^ RABL6A promotes the proliferation and survival of multiple tumor types and its increased expression is a marker of poor survival in pancreatic adenocarcinoma,^13^ breast cancer,^14,15^ and nonsmall cell lung cancer.^16,17^ Histochemical analyses of diverse patient sarcomas, including MPNSTs, showed RABL6A expression is associated with higher tumor grade and shorter time to metastasis.^18^ In NF1 patient samples, RABL6A expression is upregulated during the stepwise transformation process with significantly higher levels in MPNSTs versus benign neurofibromas (NFs).^19^ Intermediate lesions, called atypical neurofibromatous neoplasms of uncertain biological potential (ANNUBP), displayed intermediate levels of RABL6A. In MPNST cell lines, RABL6A was found to be necessary for their proliferation and survival, in part through negative regulation of the retinoblastoma (RB1) tumor suppressor pathway. These data suggested a critical role for RABL6A in driving MPNSTs. In this study, we directly examined the biological significance of RABL6A in primary MPNST development and growth using CRISPR-based models of disease lacking either *Nf1 + p53* or *Nf1 + Cdkn2a*.

## Materials and Methods

### Primary MPNST Generation and Growth Analysis

Mice were housed in the University of Iowa Animal Care barrier facility in rooms with free access to food and water and a 12 hr light-dark cycle. All mouse handling was conducted in strict compliance with the University of Iowa Institutional Animal Care and Use Committee (IACUC) policies under animal care protocol #7112074. These requirements adhere to the National Institutes of Health Guide for the Care and Use of Laboratory Animals and the Public Health Service Policy on the Humane Care and Use of Laboratory Animals. Adenoviruses containing Cas9 and sgRNAs targeting *Nf1 + Cdkn2a* or *Nf1 + Trp53* were produced as previously reported.^11^ Tumors were initiated by injecting 10 μl of CRISPR-Cas9 adenoviruses (4 × 10^6^ pfu/μl) into the left sciatic nerve of wild-type or *Rabl6* deficient C57BL/6N mice, according to previously established methods.^9,11,20^*Rabl6* knockout C57BL/6N mice were generated by the Knockout Mouse Project, as described in.^21^ Daily caliper measurements were used to assess tumor growth, as previously described,^11,20^ where tumors arise as discreet, palpable nodules. Initiation date was determined upon first measurable nodule (125–215 mm^3^ range), where the smallest individual measurement was 6 mm. Tumor volume was calculated using the formula (length × width × thickness xπ)/6. Animals were observed for signs of poor health (weight loss, ruffled fur, immobility, and abdominal rigidity). Efforts were made to minimize animal suffering. Mice were euthanized once tumor volume reached 2000 mm^3^. Tumors were harvested and split for processing by fixation in 10% neutral buffered formalin or flash-frozen in liquid nitrogen.

### Histopathological Analysis

Formalin-fixed tumors were routinely processed, paraffin-embedded, sectioned (~4 μm) onto glass slides, and hydrated through series of xylene and alcohol baths. 3,3’-diaminobenzidine (DAB, brown staining) was used as the chromogen, and Harris hematoxylin (basophilic staining) was used as the counterstain. Immunostaining for YAP and Ki67 was conducted utilizing validated protocols.^22^ Antigen retrieval was performed using citrate buffer (pH 6.0), 110–125°C for 5 min, and 20 min cool down. Secondary antibodies were obtained from Dako North America, Inc. Slides were reviewed by pathologists with expertise in human sarcoma and veterinary pathology, Drs. Tanas and Meyerholz, respectively.

### RNA Isolation and RT-qPCR

RNA was prepared from flash-frozen tumors using Qiagen RNAeasy Plus Mini Kit, and cDNA was prepared from 100–200 ng RNA using SuperScript III First Strand cDNA Preparation Kit. Diluted cDNA was used for qPCR with gene-specific primers and iQ SYBR Green Supermix reagent in a Bio-Ras CFX96 Real-Time System. qPCR cycling conditions were as follows: (1) denaturation at 95°C for 10 min, (2) 40 cycles of 95°C for 15 sec, and 60°C for 1 min. Samples were run in triplicate, and fold change in gene mRNA levels were normalized to *Gapdh* mRNA expression and computed using 2^-ΔΔCt^. Mouse gene-specific RT-qPCR primers for: *Myc* (Fwd: 5’—CCTGTACCTCGTCCGATTCC—3’, Rev: 5’—TTCTTGCTCTTCTTCAGAGTCG—3’), *Ctgf* (Fwd: 5’—TTGACAGGCTTGGCGATT—3’, Rev: 5’—GTTACCAATGACAATACCTTCTGC—3’), *Cyr61* (Fwd: 5’—GTGCAGAGGGTTGAAAAGAAC—3’, Rev: 5’—GGAGGTGGAGTTAACGAGAAAC—3’), and *Gapdh* (Fwd: 5’—GTTGTCTCCTGCGACTTCA—3’, Rev: 5’—GGTGGTCCAGGGTTTCTTA—3’).

### Western Blotting

Flash-frozen tumor chunks were lysed in RIPA buffer (50 mM Tris, pH 8.0, 150 mM NaCl, 1% Triton X-100, 0.1% SDS, 0.5% sodium deoxycholate) containing 1mM NaF, protease, and phosphatase inhibitor cocktails (Sigma, P-8340, and P-0044) and 30µM phenylmethylsulfonyl fluoride (PMSF). BCA protein assay was used to determine protein concentrations after lysis. Equal protein amounts were electrophoresed through polyacrylamide gels, and proteins were transferred onto PVDF membranes (Millipore). Membranes were blocked with 5% nonfat milk or 5% BSA in TBST (Tris-buffered saline containing Tween-20) depending on the antibody for 1hr at room temperature. Membranes were incubated with primary antibody solutions overnight at 4°C. HRP-conjugated secondary antibodies and enhanced chemiluminescence (ECL, Amersham, Buckinghamshire, UK) were used to detect proteins. ImageJ (NIH) was used to quantify protein densitometry.

### Antibodies

All antibodies were used in accordance with supplier guidelines. Antibodies used for western blotting include those specific for: c-Myc (1:1000, ab32072) from Abcam, p27 (1:1000, #3686), phospho-AKT S473 (1:1000, #4060), AKT (1:1000, #4685), and ERK1/2 (1:1000, #4695) from Cell Signaling Technology, and p53 (1:200, FL-393, sc-6243), p16 (1:200, F-12, sc-1661), phospho-ERK1/2 T202/204 (1:200, sc-7383), and β-actin (1:500, sc-8432) from Santa Cruz Biotechnology. RABL6A polyclonal antibody (1.5μg/mL) was produced in the Quelle laboratory.^13,23^ Antibodies used for IHC were specific for YAP (1:100, sc-15407) from Santa Cruz Biotechnology and Ki67 (1:200, SP6 #16667) from Abcam.

### Analyses of RABL6A Silencing in Mouse MPNST Derived Cell Lines

Cell lines were derived from terminally harvested MPNSTs in both *Nf1 + Cdkn2a* (NC) and *Nf1 + p53* (NP) targeted lesions, as described.^20^ Cells were maintained in Dulbecco’s Modified Essential Medium (DMEM) containing 10% heat-inactivated fetal bovine serum, 4 mM glutamine, and 100 µg/ml penicillin-streptomycin. To silence mouse *Rabl6a*, previously characterized pSUPER.retro based retroviruses encoding two distinct shRNAs to *Rabl6a* (called Kd1 and Kd2) were generated and infected into the NC and NP cell lines using standard procedures.^24^ Briefly, viruses were produced in human embryonic kidney (HEK 293T) cells and transduced via three repeated, sequential infections into NC and NP cells.^24,25^ Three days after infection, infected cells were seeded at equal density onto 8-well chamber slides and the next day processed for immunofluorescent analysis of centrosome amplification and multinucleation, as described.^24^ Briefly, cells were fixed in ice-cold methanol:acetone (1:1) for 10 min, washed in phosphate-buffered saline (PBS), blocked in PBS containing 3% bovine serum albumin, and stained with antibodies to γ-tubulin (Sigma-Aldrich, clone GTU-88 mouse monoclonal, 1:1000) or α-tubulin (Calbiochem, mouse monoclonal, 1:1000). After washing in PBS and staining with Alexafluor488 secondary antibodies (Molecular Probes, 1:1000), slides were treated with ProLong Antifade mounting medium (ThermoFisher) containing DAPI to stain nuclei. One hundred or more cells per sample were counted from three or more experiments using a confocal microscope (Olympus Fluoview FV3000, Waltham, MA) to quantify numbers of centrosomes per cell and multinucleation.

### Statistics

Western data were imaged by scanning densitometry and quantified by ImageJ (NIH). Values for proteins were normalized to expression of the loading control. Differences in levels of protein expression were displayed as fold change relative WT tumors. Quantified data were presented as the mean +/- SEM. All *P* values, unless otherwise specified, were obtained by Student’s *t*-test, One-way ANOVA, or Two-way ANOVA and adjusted for multiple comparisons using the method indicated in the figure legends. Overall differences between curves were assessed using generalized linear regressions. An adjusted *P* value less than 0.05 was considered statistically significant.

## Results

### RABL6A Promotes Tumor Progression, Not Initiation, in Two Primary MPNST Mouse Models

In roughly 80% of human MPNSTs, the RB1 pathway is inactivated, primarily by loss of the *CDKN2A* gene locus.^1,3,12,26^ RABL6A is a potent oncogene that has been implicated in driving MPNST pathogenesis by negatively regulating the RB1 tumor suppressor pathway.^19^ RABL6A also has functional ties to p53 signaling, another frequently dysregulated target in MPNSTs.^3^ We first discovered RABL6A as a new partner of the Alternative Reading Frame (ARF) protein,^23^ a tumor suppressor which safeguards cells against oncogenic transformation by activating p53 and other anticancer mechanisms.^27,28^ Later work suggested RABL6A inhibits p53 activity through protein-protein interactions that enhance Mdm2-mediated degradation of p53.^29^ Together, these findings implicate RABL6A in driving MPNST pathogenesis but the exact contributions of RABL6A expression to MPNST initiation and progression are not known.

**Figure 1.**
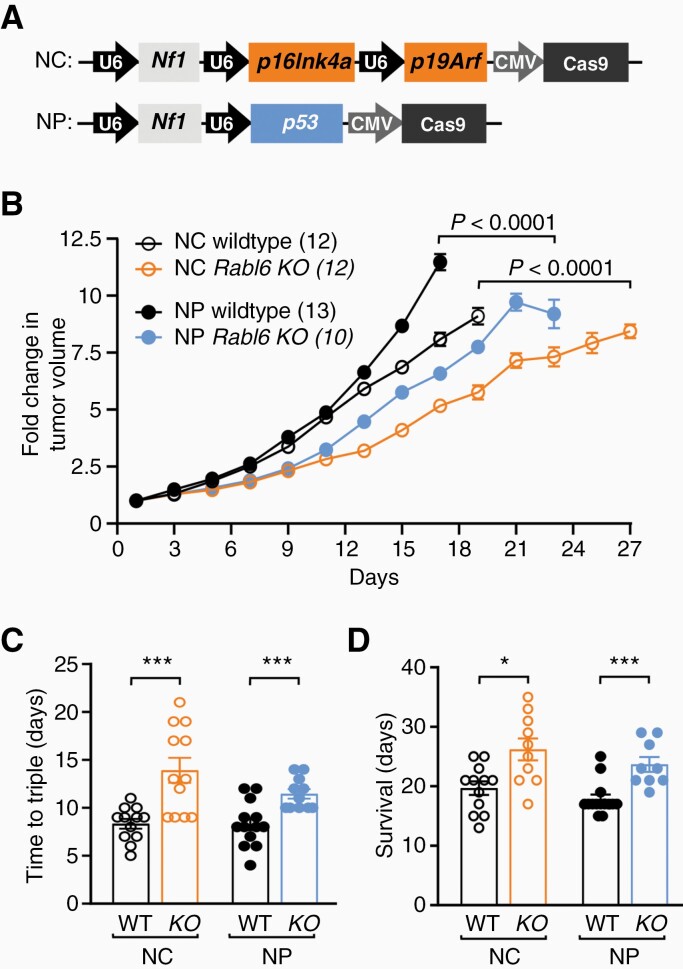
RABL6A promotes MPNST progression in both *Nf1 + Cdkn2a* and *Nf1 + p53* targeted primary MPNST mouse models. Adenoviral CRISPR-Cas9 targeting of *Nf1 + Cdkn2a* or *Nf1 + p53* was performed in the sciatic nerve of wildtype (WT) or *Rabl6* knockout (KO) C57BL/6N mice to generate primary MPNSTs. (A) Schematic of CRISPR-Cas9 targeting constructs with sgRNAs against *Nf1* with *p16Ink4a* and *p19Arf* (together comprise the *Cdkn2a* locus), designated NC, or sgRNAs against *Nf1* and *p53*, designated NP. U6 and CMV promoters are indicated. (B) Fold change in tumor volume shows mice lacking *Rabl6* display slower tumor growth kinetics in both NC and NP genetic settings. (C) Time (in days) for tumors to triple in size in WT versus *Rabl6* KO mice. (D) Survival (time to maximum 2000 mm^3^ tumor volume) of WT versus *Rabl6* KO mice. Error bars, SEM. B: *P* value determined by a generalized linear model to assess the difference between the curves. C–D: *P* value, Student’s *t*-test for KO versus WT comparisons per genotype (*, *P* < .05; ***, *P* < .001).

Therefore, we investigated the biological consequences of eliminating RABL6A during primary MPNST development. Two genetically distinct models of *de novo* MPNSTs were generated via adenoviral CRISPR-Cas9 editing of either *Nf1 + Cdkn2a* (NC) or *Nf1 + p53* (NP) in the sciatic nerve of wildtype versus *Rabl6* knockout C57BL6/N mice (Figure 1A). Those changes mimic the most common alterations seen in patient MPNSTs. Mice lacking *Rabl6* displayed significantly slower kinetics of tumor growth (Figure 1B) and time for tumors to triple (Figure 1C) in both NC and NP genetic settings. The decreased rate of tumor progression in *Rabl6* deficient mice was associated with a moderate but reproducible extension in survival in both NC and NP animals (Figure 1D). Specifically, median survival after tumor detection was increased to 26.2 and 23.6 days, respectively, in NC and NP mice relative to 19.6 and 17.9 days in matched wildtype controls (Figure 1D).

**Figure 2.**
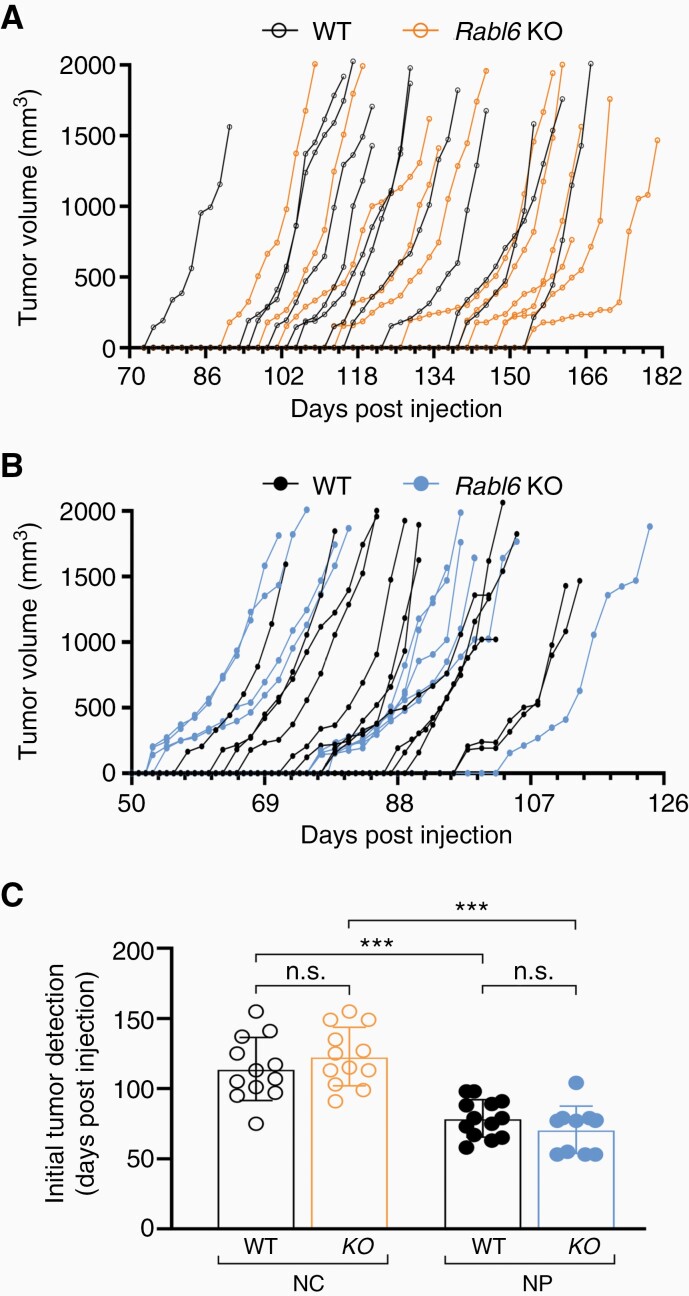
Tumor initiation is not altered by RABL6A loss in *Nf1 + Cdkn2a* and *Nf1 + p53* inactivated MPNSTs. Individual tumor initiation and growth kinetics from (A) *Nf1 + Cdkn2a* (NC) and (B) *Nf1 + 53* (NP) targeted tumors in wildtype (WT) and *Rabl6* KO mice. (C) Initial tumor detection, measured in days following CRISPR-Cas9 adenovirus injection in the sciatic nerve of each mouse within the indicated groups, shows faster MPNST formation in NP tumors. Error bars, SEM. C: *P* value, Two-way ANOVA with Tukey’s multiple comparisons test (***, *P* < .001; n.s., not significant).

Careful evaluation of tumor initiation in each condition was performed to determine if RABL6A loss influenced MPNST onset. Tumor volumes over time, from the point of initial detection until endpoint, were graphed for each mouse following injection with NC (Figure 2A) or NP (Figure 2B) adenoviruses. A striking difference in MPNST initiation, defined as when palpable lesions first become measurable, was observed between NC and NP mice (Figure 2C). The mean time to detectable tumor formation in wild-type mice following *Nf1 + Cdkn2a* inactivation was 114 days compared to only 78 days following *Nf1 + p53* inactivation. Loss of RABL6A did not affect tumor initiation in either NC or NP mice. These observations suggest RABL6A promotes MPNST progression, not initiation. Moreover, *p53* inactivation in an *Nf1*-deficient setting accelerates MPNST formation compared to *Cdkn2a* loss in the same genetic background.

### Rabl6-Deficient Tumors Display Reduced YAP Activity in a Context-Dependent Manner

**Figure 3.**
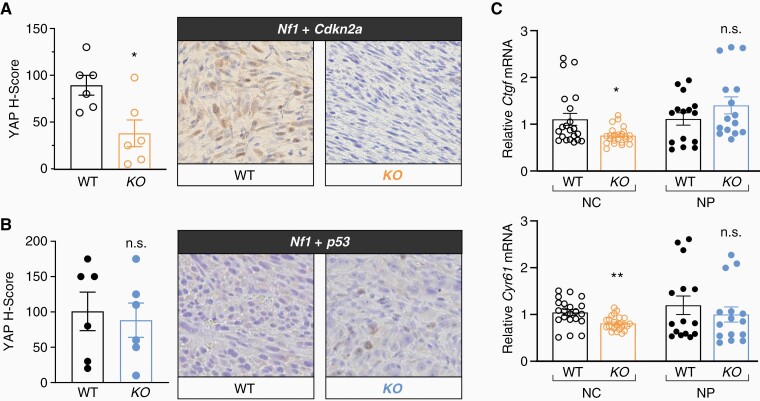
RABL6A-null tumors display reduced YAP activity in a context-dependent manner. Representative YAP IHC images (200X magnification) from WT and *Rabl6* KO mice in (A) *Nf1 + Cdkn2a* and (B) *Nf1 + p53* primary MPNSTs with H-Score quantification graphed on the left. (C) Relative mRNA levels of YAP target genes, *Ccn2* (Ctgf) (top) and *Ccn1* (Cyr61) (bottom) from WT versus *Rabl6* KO NC (*Nf1 + Cdk2na*) and NP (*Nf1 + p53*) edited tumors. YAP expression and activity (measured by downstream target expression) is decreased only in *Nf1 + Cdkn2a Rabl6* KO tumors compared to WT, whereas *Nf1 + p53* tumors remain the same. Error bars, SEM. *P* value, Student’s *t*-test for KO versus WT comparisons per genotype (*, *P* < .05; **, *P* < .01; n.s., not significant).

Recent work identified YAP (Yes-Associated Protein), a potent oncogene and transcriptional regulator involved in Hippo signaling, as a driver of MPNST pathogenesis.^1,30,31^ We recently identified a significant positive correlation between expression of YAP and RABL6A across numerous types of sarcoma, including MPNSTs.^18^ We speculated that RABL6A loss may be associated with reduced YAP expression and activity in our MPNST tumor models. Formalin-fixed, paraffin-embedded tumors were subjected to IHC for YAP, enabling analysis of its expression and nuclear localization (where it is active). RABL6A depleted NC tumors displayed reduced nuclear YAP expression (Figure 3A) whereas YAP nuclear levels were unaffected by RABL6A status in NP-generated tumors (Figure 3B). The reduced nuclear localization of YAP in NC tumors lacking RABL6A correlated with diminished mRNA levels of YAP target genes, *Ctgf* and *Cyr61*, compared to tumors from NC wildtype mice (Figure 3C). Downregulation of *Ctgf* and *Cyr61* was not observed in NP *Rabl6* KO tumors, in agreement with immunohistochemical analyses showing no differences between wildtype and *Rabl6* KO tumors. These data suggest a more important role for RABL6A-YAP signaling in *Nf1* mutant, *Cdkn2a*-null lesions compared to *Nf1* mutant MPNSTs bearing p53 alterations.

### Sustained RABL6A Loss Leads to Paradoxical Tumor Alterations Indicative of Heightened Malignancy

**Figure 4.**
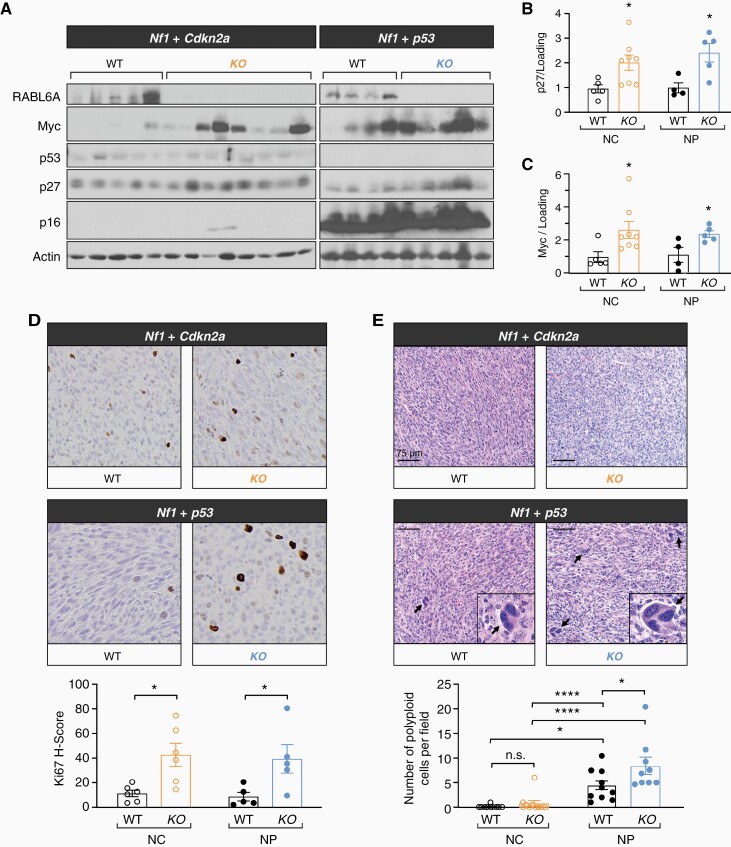
Sustained RABL6A loss leads to paradoxical molecular and pathological alterations indicative of increased malignancy. (A) Representative western blots confirming loss of RABL6A, p16, and p53 in respective conditions. *Rabl6* KO mice displayed increased p27 and Myc protein expression in both *Nf1 + Cdkn2a* (NC) and *Nf1 + p53* (NP) tumors. ImageJ quantification of (B) p27 and (C) Myc protein expression. (D) Representative Ki67 IHC images (200X magnification) and H-score quantification reveal *Rabl6* KO tumors have enhanced proliferation compared to WT tumors in both NC and NP settings. KO tumors notably also have enlarged cells. (E) Representative H&E images (scale bar, 75 µm) of the indicated tumors reveal increased cancer cell atypia with scattered enlarged cells in *Nf1 + p53* altered tumors, which was further enhanced by RABL6A loss. Arrows highlight scattered enlarged cells with large, misshapen and multiple nuclei (morphologically consistent with polyploidy cells), which are magnified in the insets. Below, the number of enlarged cells with polyploidy was quantified and averaged from 5 different fields per tumor. B,C,D,E: Error bars, SEM. B,C,D: *P* value, Student’s *t*-test for comparisons between WT and KO tumors for the indicated tumor genotype (*, *P* < .05). E: *P* value, Tukey’s multiple comparisons test (*, *P* < .05; ****, *P* < .0001).

Depletion of RABL6A suppresses *Nf1* mutant MPNST progression in both *Cdkn2a* and *p53* inactivated genetic contexts without affecting tumor initiation (Figures 1 and 2). Terminal tumors in each model were harvested and examined for molecular and histopathological alterations by western blotting, qRT-PCR, and histochemical analyses (Figure 4). Western analyses validated loss of p16Ink4a protein in NC tumors and p53 protein in NP MPNSTs, as well as RABL6A loss in *Rabl6* KO mice compared to wildtype (Figure 4A).

Prior work has shown that RABL6A promotes tumor cell proliferation and survival through multiple factors, including p27-CDK-RB1^19,32,33^ and PP2A-AKT-mTOR^34^ pathways with a role for Myc suggested by evidence that its mRNA and protein expression depend on RABL6A.^32,35^ As recently observed in human MPNSTs,^19^*Nf1* mutant mouse MPNSTs lacking RABL6A displayed increased p27 expression compared to wildtype lesions (Figure 4A–B). Surprisingly, Myc protein levels were upregulated in both NC and NP tumors lacking RABL6A (Figure 4A and 4C), despite significantly reduced *Myc* mRNA in the same samples (Supplementary Figure 1). Such data imply the involvement of post-transcriptional events leading to increased Myc protein translation and/or stabilization. Notably, levels of activated AKT (phosphorylated at S473) and activated ERK1/2 (phosphorylated at T202 and Y204) in the MPNSTs were not altered by RABL6A loss in either *Nf1 + p53* or *Nf1 + Cdkn2a* deficient settings (Supplementary Figure 2).

MPNSTs from each model were also examined for differences in tumor cell proliferation and morphology. Immunohistochemical staining for the proliferation marker, Ki67, showed that MPNSTs lacking RABL6A had significantly higher Ki67 positivity relative to wildtype tumors (Figure 4D), consistent with the observed upregulation of Myc protein (Figure 4A and 4C). Interestingly, morphologic examination of H&E stained tumor sections revealed increased atypia along with scattered enlarged cells containing multiple abnormal nuclei in NP tumors, which was increased by RABL6A loss (Figure 4E). By comparison, NC tumors had less atypia in both wildtype and *Rabl6* knockout mice (Figure 4E). Together, these results suggest a more malignant phenotype of *Nf1 + p53* inactivated MPNSTs that is accentuated by sustained absence of RABL6A.

Atypia and polyploidy (seen as enlarged cells with aberrant nuclei) are morphological features of malignancy resulting from stress and accumulated genomic instability.^36,37^ We previously showed that silencing *Rabl6* in cultured mouse embryo fibroblasts induces genomic instability in the form of centrosome amplification and multinucleation.^24^ Using mouse MPNST cell lines derived from wild-type mice with NC or NP tumors, we evaluated the effects of acute *Rabl6* knockdown on both of those aberrant events (Supplementary Figure 3A). Downregulation of endogenous RABL6A caused significant increases in enlarged, multinucleated cells (Supplementary Figure 3B) akin to polyploid giant cancer cells^36^ as well as centrosome amplification (Supplementary Figure 3C) in both NC and NP-derived MPNST cell lines. Basal levels of centrosome amplification trended higher in cultured NP cells compared to NC cells although differences were not statistically significant (Supplementary Figure 3C). These data show that acute loss of RABL6A induces significant genomic instability in cultured MPNST cells. While cells with excessive genomic abnormalities will likely die early in tumorigenesis, those with less extensive genome instability may attain a survival advantage, propagate over time, and ultimately contribute to the elevated cellular atypia, polyploidy, and malignancy in growing tumors, particularly in a p53 inactive setting (as in Figure 4E).

**Figure 5.**
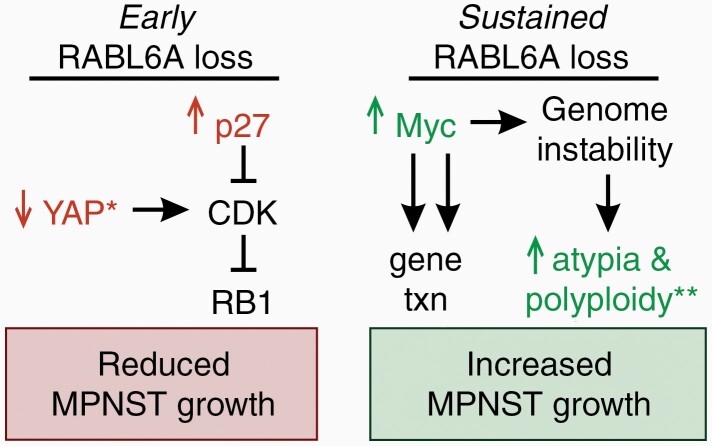
Model of molecular changes in *de novo* MPNSTs following early versus sustained RABL6A loss. RABL6A-regulated pathways proposed to mediate reduced MPNST growth in the early stages of RABL6A loss (left) versus the highly proliferative tumors that have become refractory to sustained RABL6A loss (right). Validated changes are highlighted in color. Left, initial loss of RABL6A downregulates YAP expression and signaling (*, selectively in *Nf1-Cdkn2a* inactivated tumors) along with activation of p27-RB1-mediated cell cycle arrest. Right, long-term RABL6A loss somehow leads to increased Myc protein expression, which would promote the transcription (txn) of its tumor-promoting gene targets, some of which cause genomic instability. **In *Nf1 + p53* inactivated tumors, sustained loss of RABL6A enhances tumor cell atypia and polyploidy, which are outcomes of genome instability and reflect increased malignancy.

Considered together, these data support a temporal model of how RABL6A loss influences MPNST progression *in vivo* (Figure 5). At first, the absence of RABL6A slows MPNST progression, likely due to upregulation of p27, context-dependent loss of YAP, CDK inhibition, and RB1 activation (Figure 5, left panel). Over time, however, sustained RABL6A loss provides selective pressure for molecular alterations, such as increased Myc signaling, that override the inhibitory effects of RABL6A inactivation and restore tumor cell proliferation (Figure 5, right panel). Depending on the genetic context, such as p53 inactivation and *Nf1* deficiency, Myc upregulation and consequent genomic instability may predispose tumors to enhanced atypia, polyploidy, and refractory malignant growth.

## Discussion

MPNSTs are difficult to treat cancers due to their location surrounding nerves, aggressive growth, and high mutational burden reflecting an extensive number of molecular alterations driving their development. Many factors implicated in MPNST genesis have yet to be fully tested for biological significance in disease formation. Here, we establish a critical physiological role for RABL6A in promoting NF1-associated MPNST progression.

Mounting evidence from earlier work supported the notion that RABL6A would drive MPNST pathogenesis *in vivo*. First, studies of cultured MPNST cells demonstrated RABL6A is an essential regulator of MPNST cell proliferation and survival in vitro.^19^ RABL6A was found to function, at least in part, by activating oncogenic CDK4/6 through inhibition of p27, thereby disabling RB1-mediated tumor suppression in tumor cells. Those findings, and the fact that p27 loss and RB1 inactivation are hallmark events in MPNSTs associated with worse prognosis,^38,39^ predicted RABL6A may be dysregulated in patient tumors. Indeed, RABL6A protein expression is dramatically increased in MPNSTs compared to patient-matched, benign neurofibromas.^19^ Notably, RABL6A controls many other cancer pathways besides CDK-RB1, including p53,^29,32^ AKT,^34^ and Myc.^19,32,35^ Therefore, we speculated it might have different effects on MPNST growth depending on the genetic context. Using two genetically distinct MPNST models initiated by either *Nf1 + Cdkn2a* (NC) or *Nf1 + p53* (NP) loss, we found that RABL6A is required for optimal tumor progression in both settings. However, molecular and histopathological analyses of the tumors suggest nonidentical effects of RABL6A inactivation on the biology of NC and NP tumors.

One notable difference was that YAP nuclear localization and transcriptional activity were significantly reduced in *Nf1 + Cdkn2a* tumors lacking RABL6A, but not in *Rabl6*-deficient tumors caused by *Nf1 + p53* mutation. Mechanisms underlying this difference are not currently known. However, a recent histological analysis of multiple sarcoma subtypes, including MPNSTs, revealed positive correlations between YAP and RABL6A in the patient tumors whereas no direct associations between YAP and p53 were identified.^22^ These studies suggest RABL6A may regulate YAP independent of p53 status. Future studies of the RABL6A-YAP link will reveal how biologically relevant this relationship is to MPNST genesis and whether co-targeting YAP with other RABL6A effectors, such as CDK-RB1 signaling, represents a valuable therapy regimen for *Cdkn2a*-altered MPNSTs. This is compelling because YAP activation has been implicated in a number of human cancers,^40^ most recently including MPNSTs. Wu et al. demonstrated (1) elevated YAP expression in human MPNSTs, (2) YAP hyperactivity in Schwann cells induces MPNSTs, and (3) co-targeting YAP and PDGFR pathways suppressed MPNST growth.^30^ More recently, Velez-Reyes et al. employed CRISPR-Cas9 editing of putative MPNST driver genes and identified Hippo-YAP pathway as a likely central driver of MPNST development.^31^

RABL6A loss significantly delayed the progression of MPNSTs, but molecular analyses of terminal tumors suggested its sustained absence may induce an unwanted proliferative phenotype similar to the outgrowth of drug-resistant tumors. Specifically, tumors lacking RABL6A exhibited elevated Myc protein levels as well as an increase in the proliferation marker, Ki67. The rise in Myc protein was observed despite reduced *Myc* transcript levels in the same samples, suggesting Myc may be more effectively translated and/or stabilized by post-translational modifications. These data are consistent with the idea that long-term RABL6A depletion, much like anticancer therapies, may provide selective pressure for molecular alterations that override the initial growth inhibitory effects of RABL6A loss. More mouse studies will be needed to test if Myc mediates the hyper-proliferative phenotype of *Rabl6*-depleted tumors and contributes to the heightened atypia and enlarged polyploid cells in *Nf1 + p53* inactivated tumors that are indicative of increased malignancy. This seems likely given the prominent role of Myc in aggressive cancers.^41^ It is also possible that the selective retention of YAP in *Nf1 + p53* inactivated tumors may contribute to their advanced malignant phenotype, as constitutive YAP activation drives genomic instability and represents a new potential target for MPNST therapy.^1,42^

The mechanism(s) driving Myc upregulation in tumors with persistent inactivation of RABL6A are not yet known. We speculated it may involve dysregulation of the PP2A phosphatase. PP2A is a prominent tumor suppressor whose dysregulation is seen in many human cancers and is a hallmark of cellular transformation,^43,44^ and RABL6A inhibits PP2A in another cancer type, neuroendocrine tumors.^34^ PP2A directly dephosphorylates Myc and promotes its degradation while also inhibiting AKT and ERK signaling, two downstream effectors of Ras capable of stabilizing Myc.^45^ However, tumor analyses revealed no alteration of either AKT or ERK1/2 phosphorylation at PP2A-regulated sites, suggesting other factors besides PP2A control Myc upregulation following RABL6A loss. Such factors would be expected to cooperate with hyperactive Ras (due to *Nf1* loss) to promote Myc stability and heighten its transcriptional activity.

Increased Myc may contribute in a contextual manner to the enhanced atypia caused by sustained RABL6A loss in *Nf1 + p53* inactive MPNSTs, but not in *Nf1 + Cdkn2a* mutant tumors that retain intact p53. It is well established that Myc induces multiple levels of DNA damage, chromosomal and genomic instability, and remodeling of the nuclear architecture, ultimately promoting neoplasia.^46^ Loss of p53, the “guardian of the genome,” ^47^ likewise induces significant genomic instability in cancer cells, which is enhanced by dysregulated Myc.^46^ Our findings indicate the absence of p53 is responsible for the atypia and polyploidy seen in NP tumors and suggest the upregulation of Myc following continuous RABL6A loss in those lesions further accentuates that malignant phenotype. Notably, the presence of enlarged polyploid cells (also called “polyploid giant cancer cells”), which was most prevalent in NP tumors lacking RABL6A and similarly seen after acute RABL6A silencing in cultured NC and NP cells, has been associated with higher risk of tumor recurrence and metastasis.^36^

As with all mouse tumor models, there are some limitations associated with the approaches used in this study that can be addressed in future investigations. First, the global *Rabl6* knockout mouse employed herein causes loss of RABL6A expression in nontumor host cells, such as tumor-associated fibroblasts and blood vessels, that could influence tumor progression independent of direct effects on the tumor cells. To remedy that issue, *Desert hedgehog (Dhh*)-Cre mice could be used to drive tissue-specific deletion of *Rabl6* in neural crest/ Schwann cell lineage cells. Second, co-expression of Cas9 with the sgRNAs targeting *Nf1*, *Cdkn2a*, and *p53* in the adenoviral vector means that any cell transduced with the virus could undergo gene editing. The use of a conditionally expressed Cas9 in Schwann cells would provide a more precise method of editing in the desired target tissue. Finally, it would be of interest to compare MPNST development in WT and *Rabl6* knockout mice in both *Nf1+/+* and *Nf1+/–* settings to evaluate the importance of *Nf1+/–* tissue surrounding the tumors in RABL6A driven MPNST development (as in NF1 patients). As our studies were performed in *Nf1+/+* mice, our findings provide new insight into sporadic MPNST biology by virtue of recapitulating their tumor microenvironment.

Understanding the key alterations that drive tumorigenesis is crucial for developing effective therapies. We recently reported RABL6A negatively regulates the p27-RB1 pathway to promote MPNST pathogenesis, which guided preclinical drug studies that established the efficacy of CDK-targeted therapies in suppressing MPNSTs.^19^ Here, we verify the physiological importance of RABL6A signaling in driving MPNST progression in NF1-associated tumors. We identify novel RABL6A-regulated pathways that likely contribute to tumor growth, specifically YAP and Myc signaling, and found that sustained RABL6A loss eventually yields more proliferative tumors. We liken RABL6A deficient tumors to those being treated with therapies targeting RABL6A effectors, such as CDKs. Therefore, those lesions are expected to provide a powerful platform to uncover key mediators of drug resistance. It should be informative to compare the transcriptomes and proteomes of RABL6A deficient MPNSTs with those of MPNSTs treated with CDK4/6 inhibitors, for example. Our current data suggest oncogenic YAP and Myc could be mediators of resistance that may play context-dependent roles in *Nf1 + Cdkn2a* inactivated versus *Nf1 + p53*-mutated tumors. This study provides a novel system to examine one of the most pressing clinical challenges, drug-resistant tumor growth and relapse, in cancer therapy.

## Supplementary Material

vdac047_suppl_Supplementary_Data
