# Supplementary material for: Oncogenic RABL6A promotes NF1-associated MPNST progression in vivo
Source: Neurooncol Adv. 2022 Apr 9;4(1):vdac047. doi: 10.1093/noajnl/vdac047 (PMC9092646; doi:10.1093/noajnl/vdac047)
Supplement: vdac047_suppl_Supplementary_Data [file vdac047_suppl_supplementary_data.docx]

Supplementary Figures

Kohlmeyer et al, Oncogenic RABL6A promotes NF1-associated MPNST progression in vivo

Manuscript ID: NOA-D-21-00087


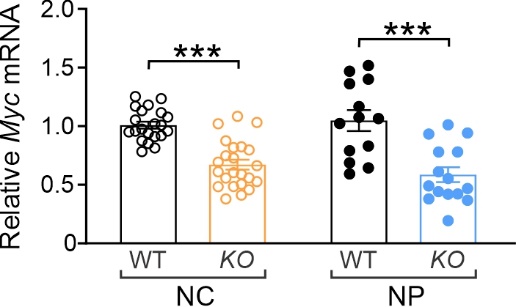


**Supplemental Figure 1.** **Tumors lacking *Rabl6* display reduced *Myc* mRNA.** Contrary to protein levels, qRT-PCR measuring *Myc* mRNA reveal downregulation at the transcriptional level in *Rabl6* KO tumors compared to wildtype (WT) in both *Nf1+Cdkn2a* (NC) and *Nf1+p53* (NP) genotypes. Error bars, SEM. *P* value, Student’s *t*-test for comparisons between WT and KO tumors for the indicated tumor genotype (***, *P* < 0.001).


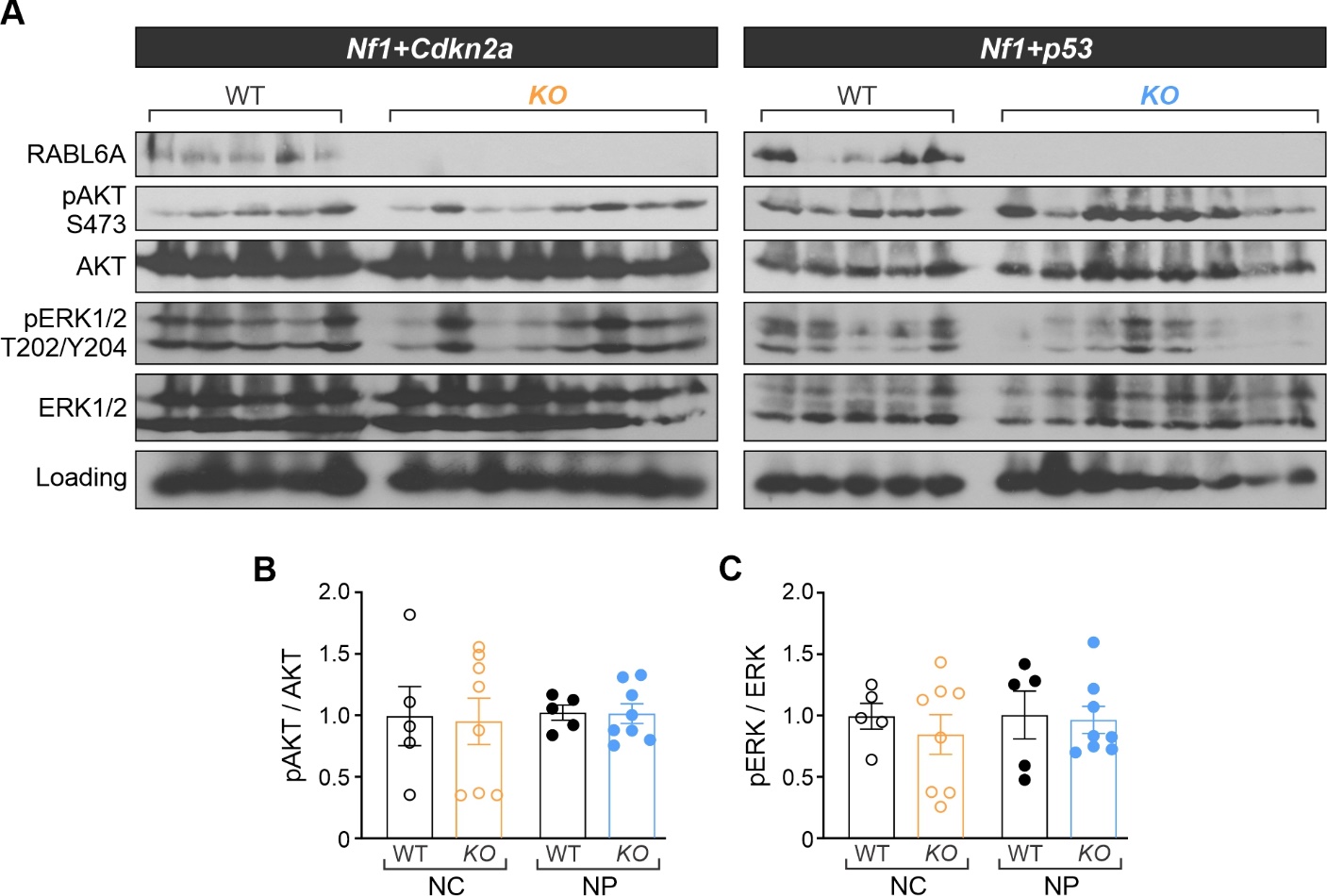


**Supplemental Figure 2. AKT-mTOR signaling, a RABL6A effector pathway in other tumor types, is not altered in de novo MPNSTs lacking *Rabl6*.** (**A**) Representative western blots confirming loss of RABL6A and evaluating phosphorylation of AKT (S473) and ERK1/2 (T202/204), established modifications indicating activation of each kinase. *Rabl6* KO mice displayed similar AKT and ERK1/2 phosphorylation levels in both *Nf1+Cdkn2a* (NC) and *Nf1+p53* (NP) tumors. ImageJ quantification of (**B**) pAKT-S473 and (**C**) pERK1/2-T202/204 relative to total protein levels. Error bars, SEM. Student’s *t*-test for comparisons between WT and KO tumors for the indicated tumor genotype revealed no significant differences.


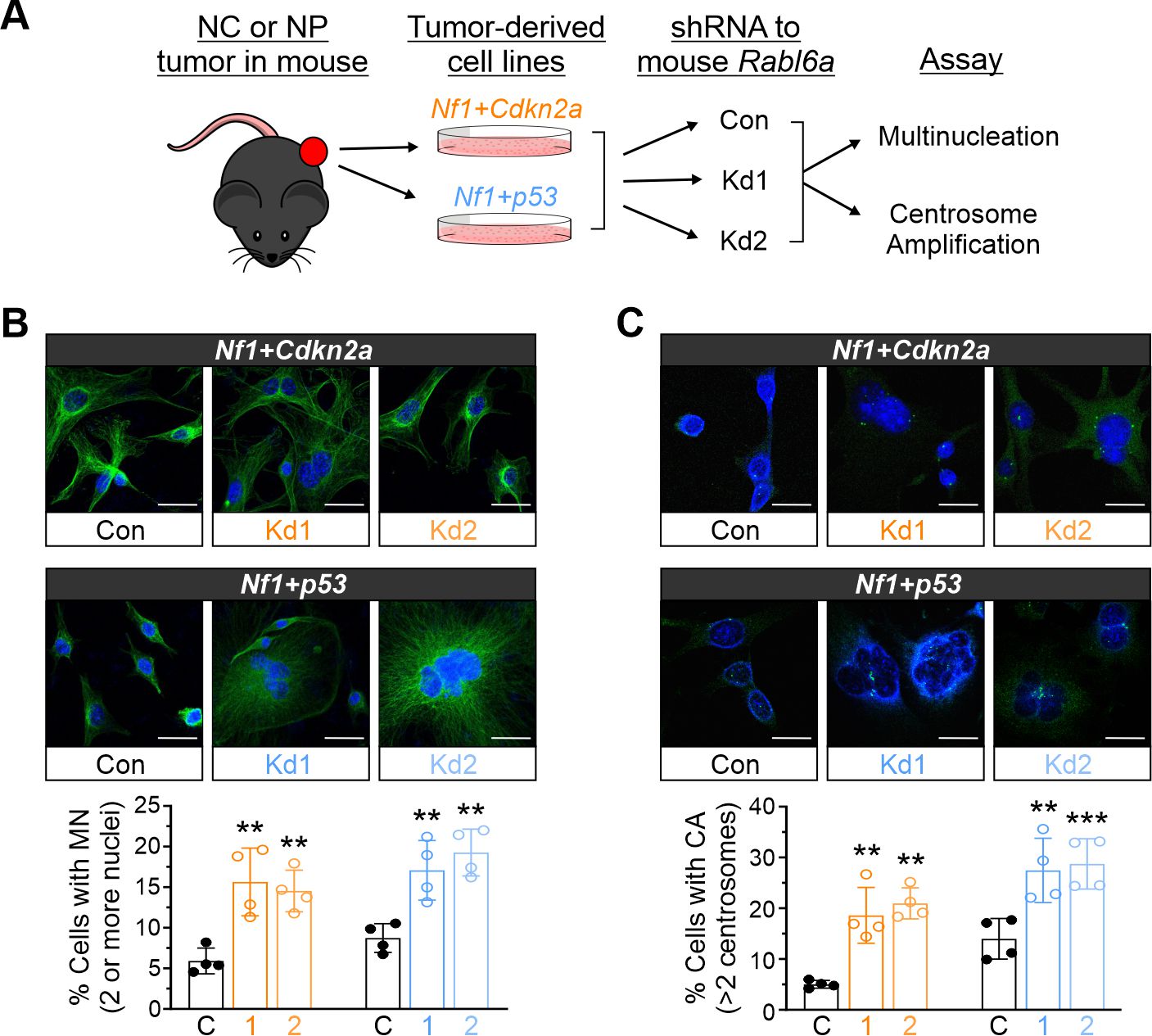


**Supplemental Figure 3.** **Acute loss of RABL6A promotes genome instability in MPNST cell lines derived from *Nf1+p53* and *Nf1+Cdkn2a* altered tumors.** (**A**) Overview depicting experimental scheme for studies of multinucleation and centrosome amplification in *Nf1+Cdkn2a* (NC) or *Nf1+p53* (NP) derived cells following *Rabl6a* silencing. (**B**) Top, confocal microscopy images of multinucleation (MN; 2 or more nuclei per enlarged cell) in control shRNA (Con, scrambled) or *Rabl6a* knockdown (Kd1 and Kd2) cells stained with alpha tubulin plus DAPI (nuclei). Bottom, quantification of percent cells with MN. (**C**) Top, confocal microscopy images of centrosome amplification (CA; > 2 centrosomes per cell) in Con, Kd1 and Kd2 cells stained with gamma tubulin plus DAPI. Bottom, quantification of percent cells with CA. B, C: Error bars, SEM. Scale bar, 25 μm. *P* value, 2-way ANOVA with Tukey’s multiple comparisons test (**, *P* < 0.01; ***, *P* < 0.001).
